# The Associations of Plasma Carotenoids and Vitamins With Risk of Age-Related Macular Degeneration: Results From a Matched Case-Control Study in China and Meta-Analysis

**DOI:** 10.3389/fnut.2022.745390

**Published:** 2022-02-11

**Authors:** Hong Jiang, Yahui Fan, Juan Li, Jiaqi Wang, Liyun Kong, Lina Wang, Zhaofang Li, Mei Ma, Xin Shi, Sijiao Liu, Jia Shi, Hailu Zhu, XiaoHong Liu, Le Ma

**Affiliations:** ^1^The First Affiliated Hospital, Xi'an Jiaotong University Health Science Center, Xi'an, China; ^2^Key Laboratory of Shaanxi Province for Craniofacial Precision Medicine Research, College of Stomatology, Xi'an Jiaotong University, Xi'an, China; ^3^School of Public Health, Xi'an Jiaotong University Health Science Center, Xi'an, China; ^4^Shaanxi Eye Hospital, Xi'an People's Hospital (Xi'an Fourth Hospital), Affiliated Guangren Hospital, Xi'an Jiaotong University Health Science Center, Xi'an, China; ^5^Key Laboratory of Environment and Genes Related to Diseases (Xi'an Jiaotong University), Ministry of Education of China, Xi'an, China

**Keywords:** carotenoids, vitamins, lutein, zeaxanthin, plasma, age-related macular degeneration

## Abstract

**Background and Purpose:**

Data from studies support a beneficial effect of carotenoids and vitamins on an age-related macular degeneration (AMD) risk. However, studies on the relations between blood levels of these nutrients and AMD are limited and provided conflicting results. The objective of this case-control study and meta-analysis was to examine whether the blood concentrations of carotenoids and vitamins were associated with the risk of AMD.

**Methods:**

A total of 164 cases of AMD and an equal number of controls are individually matched according to age and gender among the participants, who provided blood samples in the Xi'an Eye Study. Plasma carotenoids and vitamins were measured using reversed-phase high-performance liquid chromatography. Bonferroni-corrected covariate-adjusted conditional logistic regression were used to estimate AMD risk by category of these nutrients in the multivariable-adjusted model. Nine studies were identified for the meta-analysis and calculated pooled risk estimates by means of a random-effects model.

**Results:**

Plasma concentrations of examined carotenoids and vitamins were significantly lower in patients with AMD than those in controls. Plasma concentrations of examined carotenoids and vitamins were significantly lower in patients with AMD than those in controls. After a multivariate adjustment for body mass index, blood cholesterol, and other lifestyle risk factors, higher lutein/zeaxanthin content in plasma was significantly associated with a decreased risk of AMD, and the odds ratio (OR) comparing the top and bottom tertiles was 0.21 (95% CI: 0.05, 0.84; *P*_*trend*_ = 0.024). Associations for β-carotenes (OR: 0.11; 95% CI: 0.02, 0.50; *P*_*trend*_ < 0.001), and β-cryptoxanthin (OR: 0.08, 95% CI: 0.02, 0.39; *P*_*trend*_ < 0.001) were similar to that for lutein/zeaxanthin. Inverse associations were also observed for a higher level of retinol (OR: 0.14, 95% CI: 0.03, 0.61; *P*_*trend*_ = 0.006) and α-tocopherol (OR: 0.25, 95% CI: 0.06, 0.98; *P*_*trend*_ = 0.006). In the meta-analysis, a protective effect was detected for AMD among the participants with high blood lutein/zeaxanthin level (OR: 0.53, 95% CI: 0.40, 0.72, *P* < 0.001), compared to those with low level. Similar inverse associations were seen for β-carotene (OR: 0.48, 95% CI: 0.28, 0.84, *P* = 0.010), β-cryptoxanthin (OR: 0.48, 95% CI: 0.23, 1, *P* = 0.049), lycopene (OR: 0.70, 95% CI: 0.54, 0.90, *P* = 0.006) and α-tocopherol (OR: 0.50, 95% CI: 0.31, 0.81, *P* = 0.005).

**Conclusions:**

Results of the case-control study were consistent with findings from the meta-analysis, indicating that higher concentrations of carotenoids and vitamins were inversely associated with the AMD risk. Our finding supports the current notion that these nutrients are likely to affect the development of AMD and may help to refine the strategies for the prevention of age-related eye diseases.

## Introduction

Age-related macular degeneration (AMD) is a progressive degenerative disorder of the macula, an area of the retina responsible for central vision (1, 2). It is the most common cause of severe visual impairment and blindness in persons over the age of 50 years in developed countries (3). It has become a significant public health concern given that it leads to a remarkable burden on medical, economic, and society (4, 5). Even though some qualitative advances have been achieved in treating certain types of wet AMD, the therapy options for most patients with AMD are still lacking (6). With the consideration of the current demographic trends, the prevalence of AMD is predicted to continue to increase at an alarming rate in the coming decades and the projected number of people affected by AMD is expected to reach 288 million by 2040 (3). Therefore, identifying the modifiable risk factors according to the pathogenesis of AMD is of prime relevance, which would assist in developing preventive strategies and further reduce the worldwide burden of functional disability related to vision loss.

Persistent oxidative stress induced by reactive oxygen species (ROS) has been implicated as an essential contributor to the etiology of AMD (7, 8). As main non-enzymatic antioxidants in the retina, carotenoids and vitamins have been postulated to have the capacity to protect the retina against oxidative damage by scavenging free radicals and reactive oxygen-based on available evidence from *in vitro* and animal experiments (9, 10). In particular, the xanthophyll carotenoids lutein and its structural isomer, zeaxanthin, are considered as a blue light filter, which confers a protective effect against photo-oxidative damage on the neural retina (11, 12). Nevertheless, most studies to date regarding the associations of carotenoids and vitamins with age-related eye diseases have focused on dietary intake, and the findings have been inconsistent (13–15). The discrepancies in studies might be attributed to confounding effects resulting from memory errors, recall bias, as well as variations in the bioavailability of these nutrients (16–18). Plasma measurement represents an alternative approach, which could provide valid and objective assessments that are free of measurement errors that are intrinsic to self-reporting instruments. However, the results from existing studies on the circulating levels of carotenoids and vitamins and AMD risk are mixed across populations, and relevant studies pertaining to Asian populations are scarce (19–21). Moreover, evidence about the association of AMD with blood levels of these nutrients has not yet been synthesized thus far, and a meta-analysis is warranted before comprehensively evaluating the potential eye health effects of carotenoids and vitamins.

Therefore, we aimed to examine the associations between plasma concentrations of these nutrients and AMD risk among the Chinese population in a matched case-control study. Moreover, we combined our results with prior studies as part of a meta-analysis to quantitatively assess the strength of epidemiological evidence on this critical issue.

## Methods

### Study Population

The Xi'an Eye Study is a multicenter, randomized, double-masked, and placebo-controlled trial with the primary aim to evaluate the efficacy of lutein and/or fish oil supplementation in the primary and secondary prevention of AMD (ChiCTR1900028680). Potential participants were recruited consecutively through advertisements, and flyers posted in clinics and at health fairs. Participants were enrolled between November 2016 and December 2019. Information on medical history, lifestyle, dietary intake, and health-related behaviors were recorded by validated questionnaire at baseline. Each participant underwent a complete optometric or ophthalmologic examination, including the best-corrected visual acuity, slit-lamp examination, intraocular pressure, optical coherence tomography, color fundus photography, and fundus autofluorescence by the ophthalmologists who are using a standardized protocol. A fasting blood sample was taken at the time of interview.

**Figure 1:**
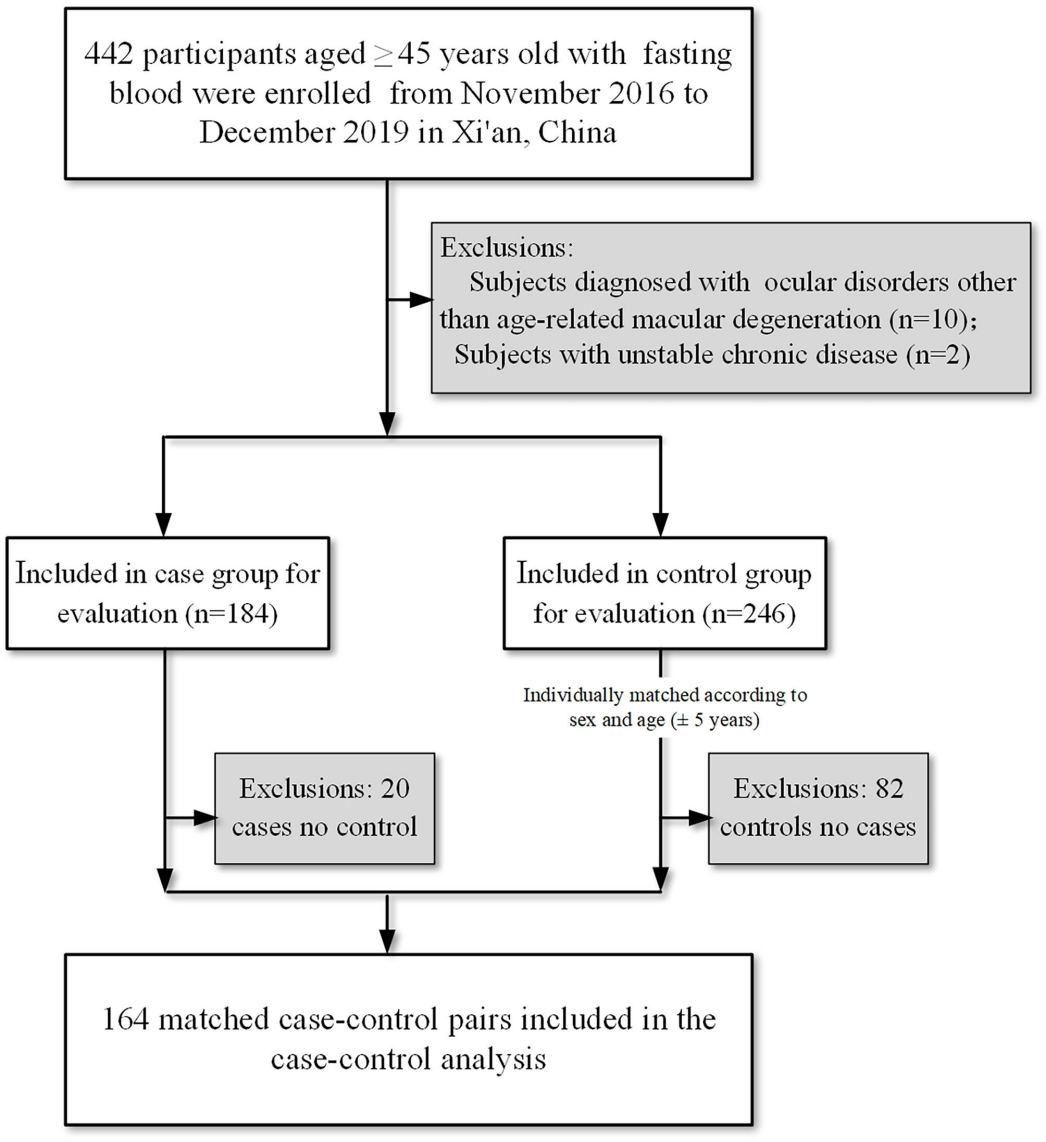
The flowchart of study participant inclusions and exclusions.

The participants included in the current investigation were aged 45 years or older, with available data for circulating carotenoids and vitamins at baseline (*n* = 442). Patients were eligible for the analysis if they were diagnosed with AMD according to the Age-Related Eye Disease Study System (22). Cases of high myopia, glaucoma, significant central lens opacities, and any other ocular disorders, such as diabetic retinopathy, branch retinal vein or artery occlusion, and central serous chorioretinopathy, were excluded. Subjects with a history of intraocular inflammation, ocular trauma, and prior intraocular surgery within 6 months were excluded. Those who had an unstable chronic illness or taking photosensitizing drugs (such as phenothiazines and chloroquine) were also excluded. The corresponding control was randomly selected among the subjects without AMD. After exclusions, cases and controls were matched on age and sex, leaving 164 pairs (164 cases and 164 controls) included for the analysis (Figure 1; Supplementary Table S1).

The study was reviewed and approved by the Human Research Ethics Committee of the Xi'an Jiaotong University Health Science Center (2014-154) and adhered to the Declaration of Helsinki's tenets for research involving human subjects. Written informed consent was obtained from each participant at enrollment.

### Ophthalmic Examination

All ophthalmologic examinations were performed in accordance with the standardized procedure by clinical technicians following pupillary dilation to obtain high-quality imaging. All photographs were reviewed by an ophthalmologist who was masked to participant information. Early AMD is defined as the presence of drusen, retinal pigmentary abnormalities (hyper-or hypopigmentation), or both. Late AMD is defined by the presence of neovascular or atrophic AMD. Neovascular AMD includes serous or hemorrhagic detachment of the retinal pigment epithelium (RPE) or retina, hemorrhages, exudates, and fibrous scar tissue under the fovea. Atrophic AMD presents as a discrete atrophic area of severe depigmentation or absence of RPE cells, characterized by the feature of the sharp border and choroidal vessels that can be easily visualized (23).

### Blood Collection and Measurement

Five-milliliter overnight fasting venous blood samples were collected into ethylenediaminetetraacetic acid vacuum tubes with anticoagulant at ambient temperature in a dimly lit room. Within 2 h of collection, the specimens were centrifuged at 3,500 g at 4°C for 10 min, and the separated plasma was transferred to 1.5 ml Eppendorf tubes immediately, then stored at −80°C freezer until analysis.

The exposures of interest were plasma lutein/zeaxanthin, β-cryptoxanthin, β-carotene, lycopene, retinol, and α-tocopherol. Plasma samples were analyzed for these carotenoids and vitamins using a reversed-phase high-performance liquid chromatography (HPLC), by the laboratory personnel who was blinded to the disease status (24). The levels of these carotenoids and vitamins were identified and determined by comparison of their specific retention times and absorption spectra with those of the authentic standards. The external standard used in the current analysis included lutein standard (Aladdin, China, ≥90% pure; CAS: 127-40-2), zeaxanthin standard (Macklin, China, ≥85% pure; CAS: 144-68-3), β-carotene standard (Aladdin, China, ≥96% pure; CAS: 7235-40-7), β-cryptoxanthin standard (Sigma-Aldrich, USA, ≥97% pure; CAS: 472-70-8), lycopene standard (Macklin, China, ≥97% pure; CAS: 502-65-8), retinol standard (Sigma-Aldrich, USA, ≥95% pure; CAS: 68-26-8), and α-tocopherol standard (Aladdin, China, ≥98% pure; CAS: 1406-18-4). Echinenone (Sigma-Aldrich, USA, ≥96% pure; CAS: 432-68-8) was employed as an internal standard to correct the quantities of post-run analytes.

Samples were prepared following a published procedure (25). The deeply frozen samples were thawed in a subdued light container at room temperature. The 250 μl plasma was precipitated with methanol and acetonitrile (250 μl; 1:2, by vol) containing the internal standard (5 μl) and was extracted into hexane. The procedure of extraction was repeated twice, and the combined supernatant was removed to a vacuum concentration system, followed by dryness under a stream of nitrogen. The dried residue was reconstituted in 100 μl of methanol, and 20 μl was injected into the HPLC system. As carotenoids are sensitive to light and oxygen, all procedures were protected from the action of light. HPLC system was equipped with a YMC-carotenoid C30 analytic column (5 μm, 250 mm ^*^4.6 mm, YMC Corporation, Kyoto, Japan) and an SPD-M20A diode-array detector (Shimadzu Corporation, Kyoto, Japan). Aliquots were run at a flow rate of 1 ml/min using Methanol (mobile phase A) and Methyl Tertiary Butyl Ether (mobile phase B) as a modifier, with the following gradient of mobile phase A [*t* (min), A%]: 0–5 min 100–95% A; 5–15 min 50% A; 15–20 min 50–40% A; and 20–23 min 40–0% A. Chromatograms were acquired at 450 nm for carotenoids and 280 nm for vitamins with the use of purified external standards. The linearity of the method was constructed by injection of the reference standard in the range of 0.005–50 μg/ml. The calibration curves of these nutrients showed a good linearity with correlation coefficients of >0.996.

### Assessment of Covariates

Information on demographic factors (age, sex), sociodemographic characteristics (educational level), lifestyle (smoking status, alcohol consumption, antioxidant supplement use, and physical activity), history of the physician-diagnosed disease (diabetes mellitus and coronary heart disease), and family history (diabetes mellitus and coronary heart disease) were collected by trained fieldworkers who were unaware of the disease status. The height and weight (light clothes without shoes) of each subject were measured with standardized protocols. Body mass index (BMI) was calculated as weight in kilograms/square of height in meters. Blood cholesterol was measured using enzymatic procedures on an autoanalyzer (Hitachi 7600-020; Hitachi, Tokyo, Japan).

### Statistical Analysis

Differences in characteristics between cases and controls were compared using Student's paired *t-*test and Wilcoxon's signed-rank test, or McNemar's tests. Participants were divided into tertiles according to the distribution of exposure concentrations of carotenoids and vitamins, whereby the lowest category was served as the reference group. A conditional logistic regression model was used to examine the associations between the circulating levels of these nutrients and AMD risk. Bonferroni correction was applied to multiple comparisons, with a two-sided *P*-value <0.0083 (α = 0.05/6), defined as statistically significant. Covariates in the current analysis were selected based on the assumption that the factors might have a biologically plausible role in either the AMD risk or a determinant of circulating carotenoids or vitamins concentration or both. Exploratory univariate analysis found that seven variables, including cigarette smoking (never, past, or current), alcohol drinking (never, or ever), educational level (less than college, or college), self-reported physical activity level (rarely/never, 1–7 h/w, or >7 h/w), BMI (continuous), blood cholesterol (continuous), and diagnosis of type 2 diabetes (yes, or no), were significant and included in the multivariate model. The significances of linear trends across the increasing categories of blood carotenoids and vitamins were evaluated by assigning the median values of each quantile as its value and fitting these continuous variables in separate regression models.

Several sensitivity analyses were conducted to assess the robustness of the results: First, we re-analyzed the associations of carotenoids and vitamins with the risk of AMD when additionally adjusted for intakes of major dietary variables. Second, we further adjusted for sunlight exposure, sun-protective gear use, and daily electronic and computerized equipment use in the models. Third, we calculated the lipid-adjusted values for carotenoids and vitamins to estimate whether the associations of these nutrients with AMD risk were independent of the concentration of cholesterol in the blood, and the associations of lipid-adjusted carotenoids and vitamins with AMD risk were analyzed. Moreover, we repeated our main analysis that included unmatched participants. All statistical analyses were done with STATA 12.0 (StataCorp, College Station, Texas, USA).

### Meta-Analysis

The PubMed, EMBASE, Web of Knowledge, and Cochrane Library were searched for articles published up to December 2021, with a combination of three groups of relevant keywords (Supplementary Table S2) (CRD42021297231). A total of 2,870 unique citations were identified after excluding duplicates. Of these, 46 relevant records were retrieved for full-text review after the screening of title and abstract. Nine studies involving 15,674 participants and 2,077 AMD cases were ultimately included in the meta-analysis (Supplementary Figure S1) (19–21, 26–31). Information on the quality of each study was independently assessed by two investigators using the Newcastle-Ottawa Scale (for cohort and case-control study), or the Agency for Healthcare Research and Quality (for cross-sectional study) (32). Random-effects models were used to synthesize the pooled risk estimates to provide more conservative results. Heterogeneity between studies was assessed with the *Q*-test and quantified with the *I*^2^ index (33). Begg's and Egger's tests and visual inspection of the funnel plots were used to test the publication bias (34). More details of the study selection and methods were described in the Methods in the Supplementary Material.

## Results

### Matched Case-Control Study

The distributions of lifestyle and health characteristics of cases and controls included in the study are presented in Table 1. Cases of AMD were more likely to smoke cigarettes, and less likely to attend higher education. Patients with AMD also tended to engage in less physical activity and less frequently reported a family history of diabetes and coronary heart disease. A history of diabetes mellitus, or coronary heart disease disorders was also more common among patients with AMD. In addition, the concentrations of examined carotenoids and vitamins were significantly lower in patients with AMD than in controls.

**Table 1:** Baseline characteristics in cases with age-related macular degeneration and control^a^.

**Baseline characteristics**	**Controls (*n* = 164)**	**AMD (*n* = 164)**	***P***
Age, years	62.50 (6.80)	62.90 (6.20)	0.37
Male, %	44.51	44.51	1.00
Han nationality, %	95.12	95.12	1.00
Cigarette smoking, %			<0.001
Never	87.20	67.68	
Past	3.66	15.85	
Current	9.15	16.46	
Alcohol consumption, %	6.71	13.41	0.06
Education level, %			<0.001
Less than college	29.27	56.66	
College	70.73	46.34	
Physical exercise, %			0.01
>7 h/w	34.15	25.61	
1–7 h/w	50.00	45.12	
Rarely/never	15.85	29.27	
Multivitamin use, %	35.98	43.29	0.04
Body mass index, kg/m^2^^b^	23.14 (21.20, 25.11)	24.06 (21.96, 26.25)	0.39
History of diabetes, %	9.15	17.07	0.05
History of coronary heart disease, %	7.32	10.98	0.33
Family history of diabetes, %	23.17	17.68	<0.001
Family history of coronary heart disease, %	29.88	17.68	<0.001

a *Data are represented as a mean value, unless otherwise indicated*.

b *Median (P25, P75)*.

Table 2 shows the risk estimates and 95% CIs of AMD risk comparing the extreme categories of blood carotenoids and vitamins concentrations. In crude analysis conditioned on matching covariates, the concentration of lutein/zeaxanthin was associated with a lower prevalence of AMD [odds ratios (ORs): 0.14, 95% CI: 0.09, 0.47; *P*_*trend*_ <0.001]. Additional multivariate adjustment for body mass index, blood cholesterol, and other lifestyle risk factors did not attenuate the associations. When comparing the top to bottom tertiles, the corresponding OR across categories of plasma lutein/zeaxanthin were 1 (reference group), 0.41 (95% CI: 0.13, 1.27) and 0.21 (95% CI: 0.05, 0.84) (*P*_*trend*_ = 0.0024), respectively. Consistent inverse associations were also observed for β-carotene, and β-cryptoxanthin. The corresponding ORs for the comparison of the extreme tertiles was 0.11 (95% CI: 0.02, 0.50; *P*_*trend*_. <001) for β-carotene, and 0.08 (95% CI: 0.02, 0.39; *P*_*trend*_ < 0.001) for β-cryptoxanthin. No significant association was found for blood lycopene concentration and AMD risk (OR: 0.27, 95 % CI: 0.07, 1; *P*_*trend*_ = 0.078). With respect to vitamins, higher concentrations in both plasma retinol and α-tocopherol concentration were inversely associated with AMD risk, with an estimated ORs of 0.14 (95% CI: 0.03, 0.61; *P*_*trend*_ = 0.006) for retinol and 0.25 (95% CI: 0.06, 0.98; *P*_*trend*_ = 0.006) for α-tocopherol when comparing the highest with the lowest category of plasma concentration.

**Table 2:** Odds ratio (95% confidence intervals) of age-related macular degeneration by tertiles of plasma carotenoids and vitamins concentration.

**Nutrient**	**Tertiles of plasma concentration**			***P_trend_^b^***
	**1^a^(ref)**	**2**	**3**	
**Lutein/zeaxanthin**				
Median, μmol/L	0.318	0.556	0.971	
No. of cases/controls	94/55	52/55	18/54	
Model 1^c^	1	0.17 (0.29, 1.06)	0.14 (0.09, 0.47)	<0.001
Model 2^d^	1	0.41 (0.13, 1.27)	0.21 (0.05, 0.84)	0.024
**β-carotene**				
Median, μmol/L	0.162	0.210	0.283	
No. of cases/controls	113/55	38/55	13/54	
Model 1^c^	1	0.31 (0.15, 0.64)	0.12 (0.05, 0.30)	<0.001
Model 2^d^	1	0.45 (0.14, 1.43)	0.11 (0.02, 0.50)	<0.001
**β-cryptoxanthin**				
Median, μmol/L	0.042	0.152	0.399	
No. of cases/controls	142/55	12/55	10/54	
Model 1^c^	1	0.08 (0.03, 0.24)	0.07 (0.02, 0.22)	<0.001
Model 2^d^	1	0.06 (0.01, 0.40)	0.08 (0.02, 0.39)	<0.001
**Lycopene**				
Median, μmol/L	0.026	0.028	0.031	
No. of cases/controls	69/55	57/55	38/54	
Model 1^c^	1	0.84 (0.45, 1.56)	0.57 (0.29, 1.11)	0.240
Model 2^d^	1	0.65 (0.22, 1.96)	0.27 (0.07, 1.00)	0.078
**Retinol**				
Median, μmol/L	3.457	4.760	6.274	
No. of cases/controls				
Model 1^c^	1	0.17 (0.07, 0.41)	0.14 (0.06, 0.35)	<0.001
Model 2^d^	1	0.25 (0.07, 0.89)	0.14 (0.03, 0.61)	0.006
**α-tocopherol**				
Median, μmol/L	32.765	50.147	71.684	
No. of cases/controls	125/55	23/55	16/54	
Model 1^c^	1	0.22 (0.10, 0.48)	0.13 (0.05, 0.32)	<0.001
Model 2^d^	1	0.12 (0.03, 0.48)	0.25 (0.06, 0.98)	0.006

*ref, reference*.

a *Reference group*.

b *P for trend over the quintile categories used the median for each quintile category as a continuous variable*.

c *Model 1: adjusted for age (years) and gender*.

d *Model 2: additionally adjusted for cigarette smoking (never, past, or current), alcohol drinking (never/ever), education level (less than college or college), self-reported physical activity level (rarely/never, 1–7 h/w, or >7 h/w), BMI (kg/m^2^), blood cholesterol (mmol/L), and diagnosis of type 2 diabetes (yes or no)*.

*P < 0.0083 was considered statistically significant after the Bonferroni correction*.

In the sensitivity analyses, the associations for individual circulating carotenoids and vitamins did not change appreciably with further adjustment for major dietary factors or sunlight exposure and personal electronic devices use (Supplementary Table S3). Sensitivity analyses, with the use of lipid-adjusted values for carotenoids and vitamins as the exposure, were consistent with our main results (Supplementary Table S3). Also, the results were largely consistent with those from the primary analyses when we include the unmatched participants (Supplementary Table S3).

### Meta-Analysis

**Figure 2:**
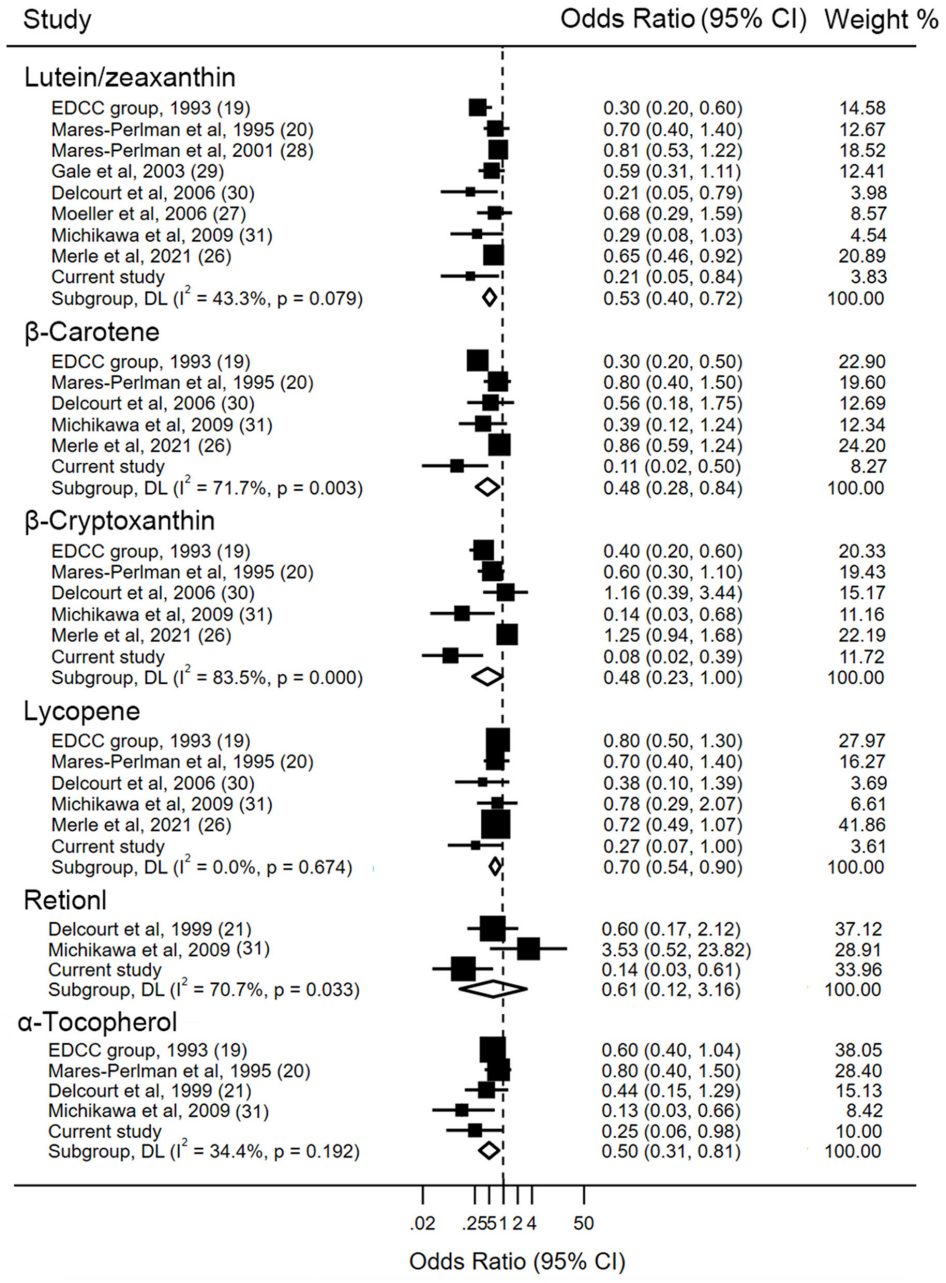
Forest plot of blood carotenoids and vitamins status and risk of age-related macular degeneration. The size of the black squares indicates the relative weight of each estimate, horizontal lines indicate 95% CIs, and diamonds indicate the synthesized odd ratio estimates with 95% CIs.

Detailed characteristics regarding each study are displayed in Table 3. The associations between levels of blood lutein/zeaxanthin and the risk of AMD were evaluated by pooling data from nine studies, and the results indicated that in comparison to those in the lowest category of blood lutein/zeaxanthin concentration, participants in the highest category had a 47% lower risk of developing AMD (OR: 0.53, 95% CI: 0.40, 0.72, *P* < 0.001; *I*^2^= 43.3%, *P*_heterogeneity_ = 0.079). Results stratified by factors yielded similar results to the main analysis (Supplementary Table S4). The pooled estimate of the OR based on six studies showed that β-carotene concentrations were associated with significantly reduced risk of AMD (OR: 0.48, 95% CI: 0.28, 0.84, *P* = 0.01; *I*^2^ = 71.7%, *P*_heterogeneity_ = 0.003). A subgroup analysis by geographic region showed that the significant inverse association between blood β-carotene levels and risk of AMD was only found among the Asians (Supplementary Table S4). Similar to the analysis for β-carotene, five studies in addition to the present analysis, assessed the circulating β-cryptoxanthin and AMD risk. The pooled estimate for the comparison of extreme categories was 0.48 (95% CI: 0.23, 1, *P* = 0.04; *I*^2^ = 83.5%, *P*_heterogeneity_ < 0.001). In stratified analyses, no significant difference was observed for any subgroups (Supplementary Table S4). Six studies contributed to the summary estimate of the association between lycopene and AMD risk. The pooled result showed that participants in the highest category of blood lycopene concentrations had a 30% lower risk of AMD (95% CI: 0.54, 0.90, *P* = 0.006; *I*^2^ = 0.0%, *P*_heterogeneity_ = 0.67). Three studies provided information on the risk of AMD and blood retinol level, and the pooled result did not show a significant association between the circulating retinol and the AMD risk (OR: 0.61, 95% CI: 0.12, 3.16, *P* = 0.557; *I*^2^ = 70.7%, *P*_heterogeneity_ = 0.03), when comparing the highest vs. lowest categories of blood retinol level. Four studies in addition to the present study investigated the association between α-tocopherol and AMD risk. The summary OR of AMD for the highest compared with the lowest category of blood α-tocopherol concentration was 0.50 (95% CI: 0.31, 0.81, *P* = 0.005; *I*^2^ = 34.4%, *P*_heterogeneity_ = 0.19) (Figure 2).

**Table 3:** Characteristics of included studies that investigated the associations of blood carotenoids and vitamins status and age-related macular degeneration.

**References, country**	**Characteristics of the study**				**Characteristics of the exposure**			**Characteristics of the participants**					**Adjustment for confounding factors**	**Study quality^c^**
	**Design**	**Study name**	**Age range (year)**	**Men (%)**	**Assay method**	**Biological sample**	**Exposure**	**Control source**	**Ascertainment method**	**No**.	**Control (*n*)**	**Cases (*n*)**		
Merle et al. (26), France	Cohort (7.6 years follow-up)	The Alienor study	≥73	36.3	HPLC	Fasting plasma	Lutein (0.30; 0.29) mmol/L; zeaxanthin (0.07; 0.07) mmol/L; β-carotene (0.75; 0.70) mmol/L; ß-cryptoxanthin (0.30; 0.29) mmol/L; lycopene (0.46; 0.41) mmol/L (mean)^b^	PBS	International classification system	609	–	54 late AMD	Sex, smoking, alcohol marital status, AMD grade at baseline, BMI, supplement use, blood draw season, diabetes, blood cholesterol, triglycerides, physical activity, AMD genetic risk score	High
EDCC Group (19), USA	Case control	EDDC	55–80	43.8	Reverse-phase HPLC	Fasting plasma	Lutein/zeaxanthin (0.25; 0.67) μmol/L; β-carotene (0.21; 0.74) μmol/L; ß-cryptoxanthin (0.09; 0.33) μmol/L; lycopene (0.19; 0.61) μmol/L; α-tocopherol (24.50; 43.39) μmol/L (median)^b^	HBS	NR	–	615	421 late AMD	Age, sex, clinic, smoking, blood cholesterol, horizontal cup-disc ratio	High
Mares-Perlman et al. (20), USA	Nested case-control	BDEM	≥43	50.0	HPLC	Non-fasting serum	Lutein/zeaxanthin (0.29; 0.29) μmol/L; β-carotene (0.38; 0.39) μmol/L; ß-cryptoxanthin (0.18; 0.19) nmol/L; lycopene (0.51; 0.51) μmol/L; α-tocopherol (33.9; 32.3) μmol/L (mean)^a^	PBS	WARMGS	–	167	167 early and late AMD	Age, blood cholesterol, beer drinking, vitamin C, vitamin E, and zinc use	High
Moeller et al. (27), USA	Case-control	CAREDS	50–79	NR	Reverse-phase HPLC	Fasting plasma	Lutein/zeaxanthin (0.15; 0.50) μmol/L (median)^b^	PBS	WARMGS	–	1.787	331 early AMD	Age, smoking, family history of AMD, diabetes and CVD, hormone replacement therapy use	High
Present study, 2021, China	Matched case-control	Xi'an Eye Study	≥45	48.6	Reverse-phase HPLC	Fasting plasma	Lutein/zeaxanthin (0.32; 0.97) μmol/L; β-carotene (0.16; 0.28) μmol/L; ß-cryptoxanthin (0.04; 0.40) nmol/L; lycopene (0.03; 0.51) μmol/L; retinol (3.46; 6.27) μmol/L; α-tocopherol (32.7; 71.68) μmol/L (median)^b^	PBS	WARMGS	–	296	164 early and late AMD	Age, sex, smoking, alcohol, education, physical activity, BMI, history of diabetes, blood cholesterol	High
Delcourt et al. (21), France	Cross-sectional	POLA	≥60	43.7	HPLC	Fasting plasma	Retinol (1.85; 2.80) μmol/L; α-tocopherol (27.2; 40.6) (median)^b^	PBS	International classification system	2,157	–	41 early and late AMD	Age, sex, smoking, education, BMI, history of hypertension, diabetes, CHD, stroke, and angioplasty	Moderate
Mares-Perlman et al. (28), USA	Cross-sectional	NHANES III	≥40	NR	Reverse-phase HPLC	Fasting plasma	Lutein/zeaxanthin (2.08; 2.15) μmol/L (mean)^b^	PBS	WARMGS	8,222	–	907 early and late AMD	Age, sex, smoking, alcohol, BMI, history of hypertension	High
Gale et al. (29), UK	Cross-sectional	the Jessop Hospital for Women	66–75	54.0	HPLC	Fasting plasma	Lutein/zeaxanthin (0.17; 0.25) μmol/L (median)^b^	HBS	WARMGS	380	–	78 early and late AMD	Age, smoking, alcohol, blood cholesterol, hypermetropic refractive error, angioplasty or coronary artery bypass	High
Delcourt et al. (30), France	Cross-sectional	POLA	≥60	NR	HPLC	Fasting plasma	Lutein/zeaxanthin (0.25; 0.56) μmol/L; β-carotene (0.27; 0.97) μmol/L; ß-cryptoxanthin (0.13; 0.47) μmol/L; lycopene (0.22; 0.71) μmol/L (median)^b^	PBS	International classification system	899	–	41 early and late AMD	Age, sex, smoking, BMI, lipid-standardized α-tocopherol, blood cholesterol	High
Michikawa et al. (31), Japan	Cross-sectional	Residents in Takasaki City	≥65	41.1	HPLC	Non-fasting serum	Lutein/zeaxanthin (0.48; 0.36) μmol/L; β-carotene (0.77; 0.41) μmol/L; ß-cryptoxanthin (0.19; 0.08) μmol/L; lycopene (0.20; 0.15) μmol/L; retinol (2.08; 2.15) μmol/L; α-tocopherol (26.00; 19.60) μmol/L (geometric mean)^a^	PBS	AREDS classification system	722	–	40 early and late AMD	Age, sex, smoking, alcohol, education, outdoor activity, BMI, history of hypertension and cataract surgery, blood cholesterol, hemoglobin A1c	High

*AREDS, The Age-Related Eye Disease Study; AMD, age-related macular degeneration; BDEM, the Beaver Dam Eye study; BMI, body mass index; CAREDS, the Carotenoids in Age-Related Eye Disease study; EDDC, the Eye Disease Case-Control study; HBS, hospital-based study; HPLC, High Performance Liquid Chromatography; PBS, population-based study; POLA, Pathologies Oculaires Liées à l'Age study; NHANES III, the Third National Health and Nutrition Examination study; NR, not reported; WARMGS, Wisconsin Age-Related Maculopathy Grading System*.

a *Exposure in controls vs. cases*.

b *Lowest vs. highest categories in all subjects*.

c *Study quality was assessed with the Newcastle-Ottawa Scale (for cohort and case-control study) or the Agency for Healthcare Research and Quality (for cross-sectional study)*.

*CI, confidence interval*.

Sensitivity analyses, that excluded one study at a time, did not change the statistical significance or the direction of the present findings, corroborating the robustness of the results. Besides, visual inspection of the funnel plots as well as formal testing with the Begg's test (all *P* > 0.05) and Egger's test (all *P* > 0.05) did not suggest evidence of publication bias (Supplementary Figure S2).

## Discussion

In the current study, higher blood concentrations of carotenoids and vitamins were associated with a lower risk of AMD. The corresponding results of the meta-analysis further confirmed most of these observed associations in the case-control study, indicating that plasma lutein/zeaxanthin, β-carotene, β-cryptoxanthin, and α-tocopherol had inverse associations with AMD risk. These findings are of potential importance for developing and refining strategies for the prevention of age-related eye diseases.

Despite a large body of evidence from observational studies, supporting potential links between dietary vitamin and carotenoid intake and AMD risk, the misclassification and the substantial measurement errors inherent in dietary assessments with the use of food frequency questionnaire (e.g., errors resulting from recall, subjective reporting, and cooking methods) are always a concern in nutritional research. Conversely, levels of carotenoids and vitamins in the blood may provide a more objective and accurate estimation of nutrient status. However, compared with studies on dietary data, little information is available on the relation between these nutrients' status and AMD. In a cross-section study among 722 participants aged 65 years or older, Michikawa et al. showed that late AMD was significantly associated with serum α-tocopherol and β-cryptoxanthin level, rather than retinol, α-, β-carotenes, lycopene, and lutein/zeaxanthin (31). The population-based Pathologies Oculaires Liées à l'Age study with 899 participants suggested that higher plasma xanthophylls were significantly associated with a reduced risk of age-related maculopathy (29). However, the finding of the carotenoids in the Age-Related Eye Disease Study showed that the prevalence of intermediate AMD was not statistically different between the highest and lowest levels of serum lutein and zeaxanthin concentration among 1,787 postmenopausal women (27). The result of the current study showed that higher carotenoid and vitamin levels were inversely associated with AMD risk, which suggested a favorable effect of increasing the circulating carotenoids and vitamins on eye health.

Several possible mechanisms, through which these carotenoids and vitamins, may include quenching singlet oxygen, scavenging reactive free radicals, and inhibition of inflammation. The retina resides in an environment involving abundant photosensitizer, continuous light exposure, high levels of oxygen tension, and polyunsaturated fatty acids, making it extremely susceptible to photochemical-mediated damage (35–37). The accumulation of toxic photoproducts and further generation of ROS photooxidative stress has been found to play an important role in disturbing lysosomal integrity. Reducing phagocytic capacity would result in structural damage and RPE apoptotic cell death (38–40). Carotenoids and vitamins are thought to be related to the antioxidant defense systems in the retina based on the precise information that they can physically quench or chemically scavenge ROS, contributing to the integrity and stability of cells and tissues (41, 42). These nutrients could upregulate the superoxide dismutase and glutathione peroxidase activity, and inhibit the intracellular ROS generation and lipid peroxidation, resulting in decreased concentrations of oxidation products in the retina (43). Decreased level of glutathione within cells has been shown previously to increase the risk of age-related cataract, a disease sharing many risk factors and pathologic mechanisms with AMD. In Sod2flox/floxVMD2-cre mice, zeaxanthin supplementation would reduce the mitochondrial oxidative stress and preserve the RPE structure and function *via* improving the expression of antioxidant genes and scaffolding protein Sqstm1 (44). Using acute glutathione loss cell model, Shang et al. found that vitamin E could reduce the formation of oxidized glutathione and simultaneously restore the cellular redox status of rabbit lens epithelial cells, which may protect against H_2_O_2_-induced cell death (45). In addition, these nutrients could also exert their effects on modulating the inflammatory response by repression of NF-κB signaling activation. Bian et al. observed a reverse in the expression of NF-κB-regulated inflammatory genes, such as MCP-1, IL-8, and CFH through activation of proteasome after supplementation of lutein or zeaxanthin, using lipofuscin-mediated photo-oxidation ARPE-19 cells (46). In an obesity-induced high-fat diet rodent model, lutein and zeaxanthin could modulate genes involved in oxidative stress and inflammation, including NF-κB and Nrf2 signaling pathways in the retina, which may contribute to ameliorating the inflammatory state of the retina (47). Furthermore, lutein and zeaxanthin have the capacity to filter phototoxic blue light based on the spectral properties, thereby possibly protecting it against photoreceptor damage produced by short wavelengths, which has been implicated in the pathogenesis of AMD (48). The beneficial effects of these nutrients on AMD were also supported by a recent analysis of the Alienor Study among 963 elderly subjects in France, which reported that plasma lutein and zeaxanthin but no other carotenoids were inversely associated with the incidence of AMD during a median of 7.6 years follow-up. These findings highlight the importance and necessity of further promoting the recommendation to increase blood levels of lutein and zeaxanthin as part of a healthy lifestyle for eye health.

It should be noted that stronger associations between levels of carotenoids and vitamins and AMD risk were observed in this study compared with those in previous studies. This discrepancy may be attributed to blood levels of these nutrients, cooking methods, or between-race variation in metabolic enzymes. In particular, blood carotenoid and vitamin concentration, in particular retinol and lutein, has been reported to be over 2 times higher in this study compared with studies in other racial or ethnic groups (such as Indian and European) (44, 49). The inconsistent associations may also be explainable by the distinct ways in which the carotenoid- and vitamin-rich foods are typically prepared. In typical Western cuisine, vegetables are usually eaten raw and uncooked, and carotenoids and vitamins in these uncooked foods are poorly absorbed (50). Chinese prefer to cook vegetables in oil to enhance taste and flavor. Stir-frying the foods in a small quantity of oil brought about an enormous increase in carotenoid bioaccessibility, the extent of increases ranging from 67 to 191% (50). Moreover, the proportion of carotenoids reaching the blood is likely to be modulated by carotenoid-specific metabolic enzymes. For example, the homozygous G allele of rs6564851 in β-carotene monooxygenase (BCMO-1) has been found to be associated with higher blood provitamin A carotenoid concentration and macular pigment optical density in humans (51–53). Results by Lietz et al. showed that Asian populations are carriers of an ~2- to 4-fold frequency of homozygous G allele than European ancestry populations, suggesting that race-related heterogeneity in enzyme related to carotenoid metabolism between ethnic origin might partially explain the observed findings (53).

Several potential limitations of our study should be considered. First, given its observational nature, it is impossible to establish a cause-effect relationship between antioxidants and AMD. Although multiple potential variables were carefully adjusted, residual or unmeasured confounding cannot be completely ruled out in any observational study, limiting the ability to make inferences about which came first. Also, as with any case-control study, the possibility of reverse causation remains a potential problem because the diagnosis of AMD might lead to changes in diet habits or lifestyle; nevertheless, our findings were unlikely driven by such bias. Apart from patients in the early stages may not be able to self-detect symptoms, the adverse effect of visual impairment may be masked by the excellent vision in another eye (54). Lee et al. showed in the 5th Korean National Health and Nutrition Examination that the average proportion of AMD awareness was only 1.45% among 7,403 participants (≥40 years old) who were ever diagnosed with AMD, almost 16-fold lower than the age-related cataract awareness rate (55). Second, measurements of exposure were only assessed at one time point, which may be less representative of the long-term variability of carotenoid and vitamin status and making it hard to fully capture the association of a time-varying of carotenoid and vitamin status on the development of AMD. However, a pilot study of repeated assessments over 3 years showed that levels of carotenoids were reasonably correlated over time (intraclass correlations ranging between 0.63 and 0.85), suggesting a single time measurement of carotenoid concentrations would represent long-term exposure with acceptable precision (56). Third, the present analysis was performed among participants who provided blood samples. Although no differences in the characteristics were observed between participants who are included and excluded from the current analysis, we cannot exclude the possibility that the associations may potentially be affected by the selection bias due to the observational nature of the present analysis. Therefore, the results from our study should be interpreted with caution. Forth, given that the present analysis was an ancillary study to the randomized controlled trial of lutein and/or fish oil supplementation for AMD, the mean age of cases was relatively low in our analysis, by comparison with the whole population, which might be relevant to bias due to health consciousness. Participants in our study may tend to be more health-conscious and more likely to seek medical help for visual impairment. Therefore, the present analysis might be affected by the inherent selection biases of the trial, which might influence the characteristics of the participants and limit the generalizability of the findings. Future research consisting of a broader representative population is warranted.

## Conclusions

In summary, the present study supports a protective effect of higher concentration of carotenoids and vitamins in blood against AMD risk, which provides further evidence of the associations between carotenoid and vitamin status and the risk of age-related eye problems. Further randomized clinical trials are necessary for Asians to confirm such associations and to provide the most reliable direct information to base public health recommendations for age-related eye disease prevention by nutritional supplementation with carotenoids and vitamins.

## Data Availability Statement

The raw data supporting the conclusions of this article will be made available by the authors, without undue reservation.

## Ethics Statement

The studies involving human participants were reviewed and approved by the Human Research Ethics Committee of the Xi'an Jiaotong University. The patients/participants provided their written informed consent to participate in this study.

## Author Contributions

LM, XL, HJ, and YF conceived the study and design. HJ, YF, and JL conducted the study, managed the project, and participants. JW, LK, and ZL helped in conducting the laboratory measurement and data interpretation. MM, XS, SL, JS, and HZ performed the statistical analysis. HJ, YF, and LM drafted the original manuscript and reviewed and revised the draft of the manuscript. All authors approved the final manuscript for submission.

## Funding

This study was partially supported by grants from the National Natural Science Foundation of China (NSFC-82022062; NSFC-81973025; and NSFC-81473059); Nutrition Science Research Foundation of BY-HEALTH (TY0181101); New-star Plan of Science and Technology of Shaanxi Province (2015LJXX-07); the Nutrition Research Foundation Fund of the Chinese Nutrition Society-DSM Special Research Foundation (CNSDSM2016-041); and the Fundamental Research Funds for the Central Universities (qngz2016004 and xzy032019008). The funders had no role in the study design, implementation, analysis, decision to publish, or reparation of the manuscript.

## Conflict of Interest

The authors declare that the research was conducted in the absence of any commercial or financial relationships that could be construed as a potential conflict of interest.

## Publisher's Note

All claims expressed in this article are solely those of the authors and do not necessarily represent those of their affiliated organizations, or those of the publisher, the editors and the reviewers. Any product that may be evaluated in this article, or claim that may be made by its manufacturer, is not guaranteed or endorsed by the publisher.
