# Supplementary material for: The Associations of Plasma Carotenoids and Vitamins With Risk of Age-Related Macular Degeneration: Results From a Matched Case-Control Study in China and Meta-Analysis
Source: Front Nutr. 2022 Feb 11;9:745390. doi: 10.3389/fnut.2022.745390 (PMC8873933; doi:10.3389/fnut.2022.745390)
Supplement: Supplementary file 1 [file Data_Sheet_1.pdf]

frontiers

Supplementary Material

Table S1 | Comparison of baseline characteristics of participants included and excluded from the analyses [a]

**Baseline characteristics**	**Participants included in the present study**	**Participants excluded from the present study**	***P***
Participants, n	328	114	
Age, y	62.70 (6.50)	63.96 (9.65)	0.15
Male, %	44.51	44.24	0.96
Cigarette smoking, %			0.68
Never	77.44	79.82	
Past	12.80	10.54	
Current	9.76	9.64	
Alcohol consumption, %	10.06	9.73	0.90
Education level, %			0.76
College	58.54	60.18	
Less than college	41.46	39.82	
Physical exercise, %			0.90
> 7 h/w	29.88	30.97	
1-7 h/w	47.56	45.13	
Rarely/never	22.56	23.89	
Multivitamin use %	39.63	34.21	0.14
Body mass index, kg/m^2 [b]	23.59 (21.56, 25.90)	23.15 (20.41, 25.15)	0.44
History of diabetes, %	13.11	10.61	0.49
History of coronary heart disease, %	9.15	9.73	0.85
Parental history of diabetes, %	20.43	17.54	0.24
Parental history of coronary heart disease, %	23.78	25.66	0.69

[a] Data are represented as a mean value, unless otherwise indicated.

[b] Median (P25, P75)

Table S2 | Search strategy in PubMed, Embase and Web of Science.

1.Antioxidants
antioxidants OR carotenoid OR lutein OR zeaxanthin OR “β-carotene” OR “α-carotene” OR “β-cryptoxanthin” OR lycopene OR “vitamin A” OR “vitamin E” retinol OR “α-tocopherol” OR retinoids
2.AMD
“age-related macular degeneration” OR “neovascular age-related macular degeneration” OR “age-related maculopathy” OR “choroidal neovascularization” OR “geographic atrophy” OR “macular degeneration” OR “drusen”
3. Source
“serum” OR “plasma” OR “blood” OR “circulat*”

Methods

Studies were considered for inclusion if they were published as observational studies, full-text original articles, and presenting risk estimates of odds ratio, hazard ratio, or relative risk with 95% confidence interval for the highest compared with lowest categories of blood carotenoids and vitamins levels. In the case of multiple reports originating from the same study population, data from only the one with the largest sample and most-applicable information was selected, or if similar, the most recent or informative.

Two investigators (JH and FYH) reviewed selected articles and extracted information from the studies separately using a predefined data-collection form, and any disagreements were resolved by discussion. When a study provided risk estimates with different degrees of statistical adjustment for confounding, the maximally adjusted estimate was chosen. Studies that reported risk estimates by sex or other subgroups separately were combined using a fixed effects model before inclusion in the overall analysis.

Random-effects models, which consider both within- and between-study variation, were used to synthesize the pooled risk estimates to provide more conservative results. Subgroup and meta-regression analyses subdivided by geographic region and study design were performed to investigate possible source of heterogeneity. Stata version 12.0 (StataCorp, College Station, Texas) was used for all data analyses.

TABLE S3 | Sensitivity analyses for the association between plasma carotenoids and vitamins concentration and age-related macular degeneration [a,]

	Tertiles 1	Tertiles 2	Tertiles 3	*P* trend
**Adjustment for major dietary factors**				
Lutein/zeaxanthin	1	0.39 (0.12, 1.24)	**0.21 (0.05, 0.84)**	0.024
β-carotene	1	0.46 (0.14, 1.47)	**0.10 (0.02, 0.48)**	**<0.001**
β-cryptoxanthin	1	**0.06 (0.01, 0.41)**	**0.08 (0.02, 0.39)**	**<0.001**
Lycopene	1	0.58 (0.19, 1.80)	0.26 (0.07, 1.00)	0.084
Retinol	1	0.26 (0.07, 0.94)	**0.14 (0.03, 0.62)**	**0.006**
α-tocopherol	1	**0.12 (0.03, 0.52)**	0.24 (0.06, 0.99)	**0.006**
**Adjustment for sunlight exposure and use of personal electronic devices**				
Lutein/zeaxanthin	1	0.42 (0.12, 1.41)	**0.17 (0.04, 0.85)**	**<0.001**
β-carotene	1	0.47 (0.14, 1.61)	**0.11 (0.02, 0.50)**	**<0.001**
β-cryptoxanthin	1	**0.05 (0.01, 0.44)**	**0.07 (0.01, 0.38)**	**<0.001**
Lycopene	1	0.53 (0.16, 1.76)	0.22 (0.05, 1.01)	0.036
Retinol	1	**0.22 (0.05, 0.88)**	**0.13 (0.03, 0.62)**	<0.001
α-tocopherol	1	**0.12 (0.03, 0.52)**	0.23 (0.05, 1.00)	<0.001
**Ratio of nutrients to cholesterol**				
Lutein/zeaxanthin	1	0.64 (0.18, 2.09)	0.28 (0.06, 0.99)	**0.005**
β-carotene	1	**0.13 (0.03, 0.64)**	**0.08 (0.01, 0.45)**	**<0.001**
β-cryptoxanthin	1	0.27 (0.06, 1.21)	**0.12 (0.03, 0.47)**	**<0.001**
Lycopene	1	0.35 (0.08, 1.50)	0.18 (0.02, 1.51)	0.010
Retinol	1	0.29 (0.07, 1.12)	**0.20 (0.05, 0.77)**	**0.006**
α-tocopherol	1	**0.20 (0.04, 0.93)**	**0.13 (0.03, 0.57)**	**0.006**
**Unmatched case-control analysis**				
Lutein/zeaxanthin	1	0.54 (0.28, 1.04)	**0.19 (0.09, 0.44)**	**0.005**
β-carotene	1	**0.44 (0.22, 0.85)**	**0.14 (0.06, 0.33)**	**0.006**
β-cryptoxanthin	1	**0.13 (0.06, 0.29)**	**0.11 (0.05, 0.28)**	**<0.001**
Lycopene	1	0.97 (0.51, 1.85)	0.52 (0.25, 1.00)	0.012
Retinol	1	**0.21 (0.10, 0.44)**	**0.12 (;0.05, 0.28)**	0.036
α-tocopherol	1	**0.13 (0.06, 0.28)**	**0.19 (0.08, 0.42)**	0.036

[a] Estimates are calculated in conditional logistic regression models, adjusted for age (years), gender, cigarette smoking (never, past, or current), alcohol drinking (never/ever), self-reported physical activity level (rarely/never, 1-7 h/w, or >7 h/w), BMI (kg/m^2), blood cholesterol (mmol/L), and diagnosis of type 2 diabetes.
P<0.0083 were considered statistically significant after the Bonferroni correction and indicated in bold.

Table S4 | Subgroup analyses examining the association between blood carotenoids and vitamins status and age-related macular degeneration.

Exposure	n	OR (95% CI)	I^2	P^a	P^b
**Lutein/zeaxanthin**	9	0.53 (0.40, 0.72)			
Geographic location					0.29
Europe	3	0.58 (0.40, 0.85)	58.6	0.06	
USA	4	0.58 (0.36, 0.95)	0.0	0.90	
Asia	2	0.25 (0.10, 0.65)	0.0	0.74	
Study design					0.68
Cohort	1	0.65 (0.46, 0.92)	NA	NA	
Nested case-control	1	0.70 (0.37, 1.31)	NA	NA	
Case-control	3	0.37 (0.20, 0.68)	36.1	0.21	
Cross-sectional	4	0.55 (0.33, 0.92)	43.0	0.15	
**β-Carotene**	6	0.48 (0.26, 0.84)			
Geographic location					0.55
Europe	2	0.83 (0.58, 1.17)	0.0	0.48	
USA	2	0.48 (0.18, 1.24)	82.5	0.02	
Asia	2	0.23 (0.07, 0.70)	35.7	0.21	
Study design					0.61
Cohort	1	0.86 (0.59, 1.24)	NA	NA	
Nested case-control	1	0.80 (0.41, 1.55)	NA	NA	
Case-control	2	0.25 (0.11, 0.54)	27.6	0.24	
Cross-sectional	2	0.47 (0.21, 1.06)	0.0	0.66	
**β-Cryptoxanthin**	6	0.48 (0.23, 1.00)			
Geographic location					0.31
Europe	3	1.24 (0.94 1.65)	0.0	0.35	
USA	2	0.47 (0.31, 0.72)	0.0	0.61	
Asia	2	0.10 (0.04, 0.31)	0.0	0.61	
Study design					0.47
Cohort	1	1.25 (0.94, 1.67)	NA	NA	
Nested case-control	1	0.60 (0.31, 1.15)	NA	NA	
Case-control	2	0.21 (0.04, 0.96)	74.8	0.05	
Cross-sectional	2	0.44 (0.06, 3.44)	78.9	0.03	

AMD, age-related macular degeneration; CI, confidence interval; NA, not applicable (because only 1 study); OR, odds ratio; USA, the United States of America.

[a] *P* for heterogeneity within each subgroup.

[b] *P* for heterogeneity between subgroups with a meta-regression analysis.

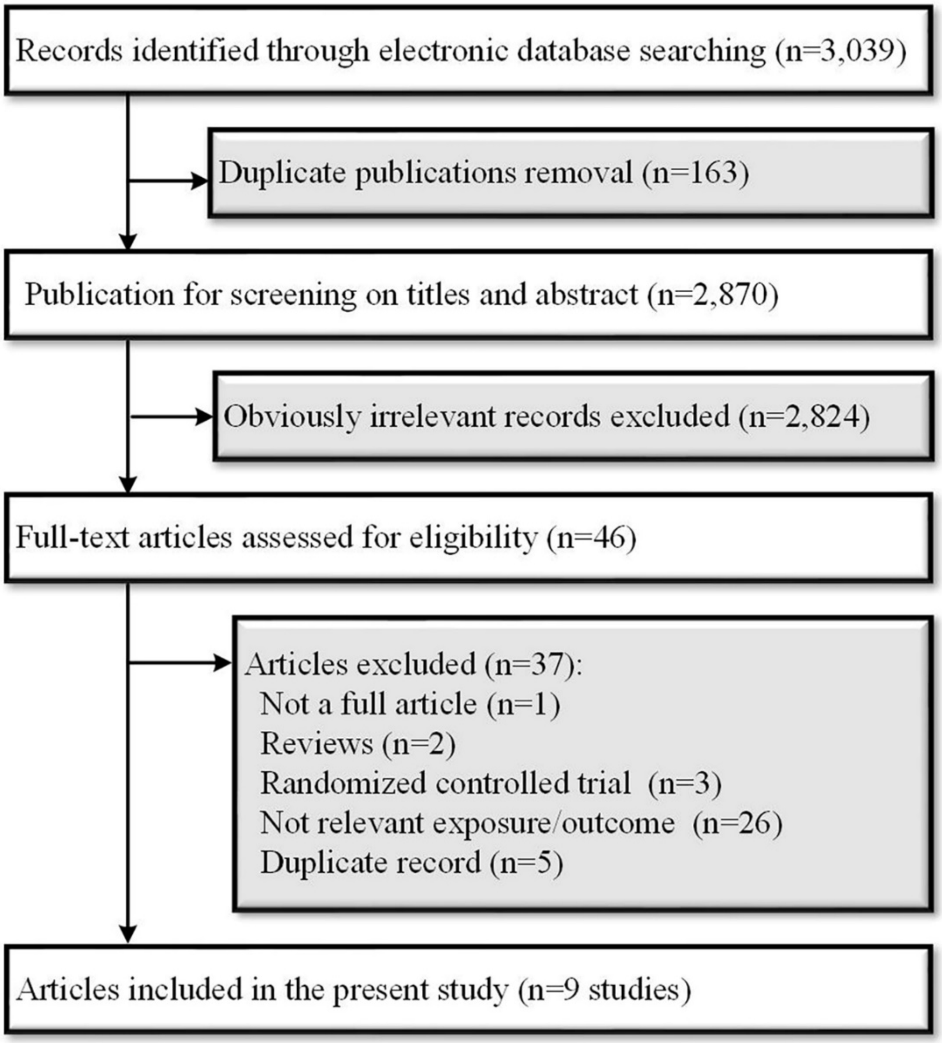

Figure S1 | Flow chart of study selection

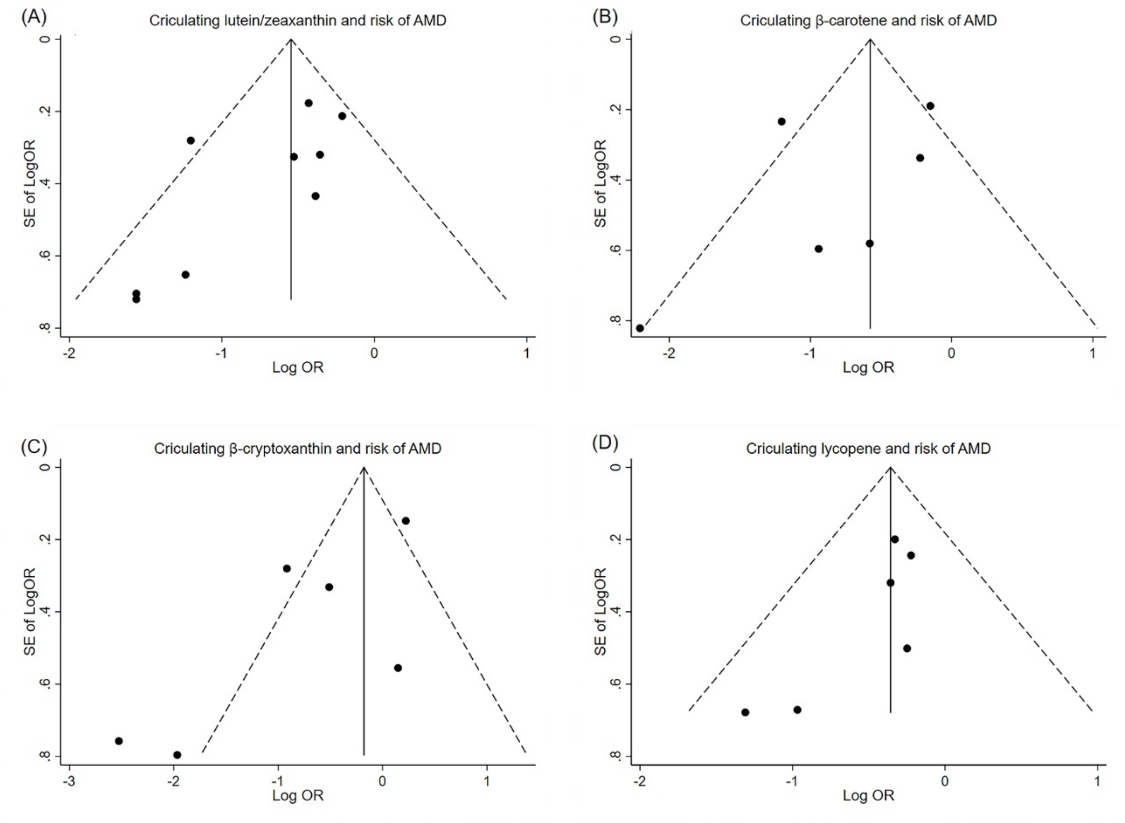

Figure S2 | Funnel plots with 95%CI of circulating lutein/zeaxanthin (A), β-carotene (B), β-cryptoxanthin (C), and lycopene (D) and risk of early age-related macular degeneration. OR, odds ratio; SE, standard error.
